# From Super-Enhancer Non-coding RNA to Immune Checkpoint: Frameworks to Functions

**DOI:** 10.3389/fonc.2019.01307

**Published:** 2019-11-22

**Authors:** Manqing Wu, Jun Shen

**Affiliations:** State Key Laboratory for Oncogenes and Related Genes, Key Laboratory of Gastroenterology & Hepatology, Division of Gastroenterology and Hepatology, Ministry of Health, School of Medicine, Shanghai Cancer Institute, Shanghai Institute of Digestive Disease, Ren Ji Hospital, Shanghai Jiao Tong University, Shanghai, China

**Keywords:** super-enhancer, non-coding RNA, cell identity, immune checkpoint, cancer, autoimmune disease

## Abstract

Super-enhancers (SEs) are clusters of enhancers that play a key role in regulating genes that determine cell identity. Enhancer RNAs (eRNAs) are non-coding RNAs transcribed from enhancers that function to promote the enhancer's functions via multiple mechanisms, such as recruiting transcription factors to specific enhancers, promoting enhancer-promoter looping, directing chromatin accessibility, interacting with RNA polymerase II and facilitating histone acetylation. Understanding how super-enhancer RNAs (seRNAs) contribute to specific gene regulation has thus become an area of active interest. Immune checkpoint deregulation is one of the key characteristics of tumors and autoimmune diseases, and is also closely related to cell identity. Recent studies revealed a potential pathway for seRNA's involvement in regulating the expression of immune checkpoints. The present study reviews the current knowledge of eRNA function, immune checkpoint blockage mechanism, and its effect. In addition, for the first time, we explore the direct and indirect roles of seRNAs in regulating immune checkpoint expression in cancer and autoimmune diseases.

## Introduction

The identification of substantial amounts of non-protein coding transcripts and their versatile functions is one of the most striking findings of contemporary genomic research. Only ~2% of the transcribed human genome is accounted for by protein coding exons, thus non-coding RNAs constitute the majority of transcripts (1). Long non-coding RNAs (lncRNAs) are non-coding RNAs that are longer than 200 nucleotides, which play a significant role in the regulation of gene expression, splicing, translation, and epigenetic regulation.

Enhancers are DNA elements of a few hundred base pairs in length that are characterized by acetylation. Enhancers interact with transcription factors (TFs) and promoters. A mammalian cell contains thousands of active enhancers and ~1 million active enhancers have been found in all human cells (2). Super-enhancers (SEs), also known as stretch enhancers, are regions where multiple enhancers are clustered together. They exert more potent effects than typical enhancers and are associated with genes that are involved in determining cell identity in both the physiological and pathological state. Cell identity genes are a cluster of functionally interconnected genes that jointly establish the unique phenotype of a given cell type on epigenomic, transcriptomic, proteomic, and metabolomic level. For instance, *NPC1L1, APOC3*, and *LCT* in enterocytes, *FOXP3, CTLA1*, and *IL2RA* in T regulatory cells (3, 4). *OCT4, NANOG*, and *PRDM14* are cell identity genes of ESC, they encode core transcription factors of embryonic stem cell (ESC) (5). These genes function together to enable expression of genes necessary to maintain its ESC pluripotency. The suppression of their expression leads to loss of pluripotency and self-renewal ability in ESC. Super-enhancers are required for cell type-specific processes and are linked with disease-associated genomic variations. Enhancer RNAs (eRNAs), another marker of active enhancers, are a novel species of non-coding RNA molecules that are transcribed from enhancer regions. Two types of eRNAs have been identified, comprising short, bi-directional and non-polyadenylated eRNAs and long, unidirectional, and polyadenylated eRNAs. The exact function of eRNAs is not clearly understood, and it has been hypothesized that eRNAs are transcription noise that do not contribute to gene expression (6). However, recent findings suggested that at least some eRNAs have a role in enhancer function by recruiting TFs to specific enhancers, promoting enhancer-promoter looping, directing chromatin accessibility, interacting with RNA polymerase II (RNAP II), and stimulating histone acetylation (7–12). Research into enhancers has expanded over the last decade and the biological function of enhancers has become increasingly clear. However, the exact function and mechanism of eRNAs are currently under investigation.

The immune system comprises innate and adaptive immunity. Immune checkpoints, consisting of co-stimulatory checkpoints and co-inhibitory checkpoints, are vital for the maintenance of self-response and prevention of autoimmunity. They are paired molecules that act as a double check before the stimulation or inhibition of an immune response. Immune checkpoints are expressed in a tissue or cell subset-specific manner. The application of immunotherapy in a wide variety of cancers has led to significant tumor shrinkage and improved clinical outcomes in patients by revitalizing the anti-tumor immune response (13, 14). The mostly widely studied inhibitory checkpoints are programmed cell death receptor-1 (PD-1), programmed cell death ligand (PD-L1), and cytotoxic T lymphocyte-associated molecule-4 (CTLA-4).

Recent studies have shown that SEs play key roles in determining cell identity in both healthy and pathological states. Over 25,000 enhancers were identified as deferentially activated in renal, breast, and prostate tumor cells, as compared with normal cells (15). This suggested a potential network between malignancy and enhancer activity. In addition, SEs are located at oncogenes and other genes that are essential for tumor pathogenesis in cancer cells, indicating their possible utility as biomarkers for tumor-specific pathologies (2). Considering the notion that evading immune destruction as a hallmark of malignancy, it is suspected that SEs in immune cells may be involved in the regulation of inhibitory checkpoint expression (16). In this review, we summarize the current understanding of eRNA function, their mechanism of action, and immune checkpoints. Then, we focus on the crosstalk between eRNA and immune checkpoints in pathological stages. A better understanding of the link between SEs, eRNAs, and immune checkpoints, may lead to eRNAs being developed as potential markers or therapeutic targets in the future.

## Super Enhancer Non-Coding RNA

**Figure 1:**
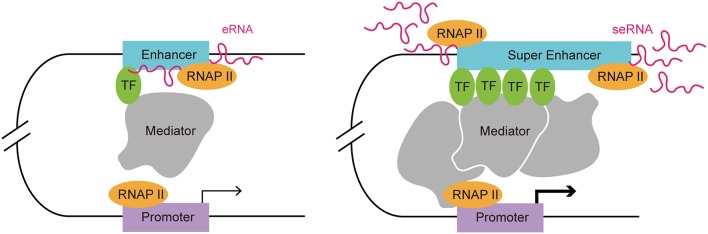
Comparison of a typical enhancer and super enhancer. Compared with typical enhancers, super enhancers are enriched with more transcription factors, mediators, and RNAP II. Hence, the transcription activity at a super enhancer is usually higher than at a typical enhancer. Functionally, super enhancers have a higher potential to promote target cell-identity-related gene transcription.

Enhancers are often occupied by multiple signature TFs. The typical chromatin signature of enhancers includes a high H3K4me1 to H3K4me3 ratio, histone H3 lysine 27 acetylation, P300 acetyltransferase binding, CREB binding protein (CBP) binding, mediator complex subunit 12 binding, and a high sensitivity to nucleases (17–22). A typical enhancer activates its target gene transcription via its *cis*-acting function along with interactions with the promoter and multiple TFs, including Yin-Yang 1 (YY1) and myogenic differentiation 1 (MYOD) (9, 23). Enhancers can exert their function in an orientation and distance-independent manner, being capable of targeting both upstream and downstream genes (24). RNAP II occupation at some enhancers leads to the transcription of eRNAs, which is considered as another hallmark of an active enhancer (12). SEs are tissue specific regulatory regions of DNA consisting of clusters of enhancers. In various murine cell types (macrophages, Th cells, pro-B cells, embryonic stem cells, and myotubes), SEs and their target genes, which encode cell-type specific TFs, have been identified (20). By investigating the distribution of disease-associated DNA sequence variation in enhancers and SEs in human cells and tissues, Hnisz et al. found that trait-associated single-nucleotide polymorphisms (SNPs) were highly enriched in SEs, indicating their potential disease-associated role (2). In addition, SEs are characterized by specific histone modifications and they bind with a higher level of mediators, nipped-B-like protein, P300, chromodomain-helicase-DNA-binding protein 7 (CHD7), Bromodomain-containing protein 4 (BRD4), kruppel-like factor 4, estrogen-related receptor beta, and cohesin compared with typical enhancers (2, 20). The level of histone modifications H3K27ac and H3K4me1 at SEs exceeded those at typical enhancers significantly (20). Moreover, RNAP II is clustered at SEs at a greater density than at typical enhancers, resulting in a higher level of super-enhancer RNA (seRNA) production (25) (Figure 1).

RNAs transcribed from enhancers can be classified as short, bi-directional, and non-polyadenylated eRNAs, and long, unidirectional, and polyadenylated eRNAs. The majority of seRNAs are capped and polyadenylated RNAs (25). This feature makes seRNAs more stable and capable of having a wider effect in physiological and pathological conditions. eRNAs are transcribed by the binding of RNAP II to enhancer DNA in various types of cells, such as macrophages, neurons, keratinocytes, and breast cancer cells (26, 27). It was proposed that TFs that are bound at enhancers interact directly or indirectly with the promoter via a cofactor to exert a stimulatory effect on RNAP II. Upon this dynamic interaction, RNAP II and its accessory effectors come close to enhancer DNA, resulting in initiation of eRNA transcription (28).

The exact function of eRNAs is incompletely understood; nevertheless, evidence suggests that at least some eRNAs play an active part in the regulation of enhancer activity and gene expression. Nicholas et al. found that the synthesis of eRNAs precedes the transcription of target gene transcription in lipopolysaccharide-activated macrophages (29). This indicated that eRNAs might be associated with transcription activation of target gene. The transcript level of the majority eRNAs correlates highly correlated with the mRNA expression level of the nearby target gene, suggesting an activating function in promoting mRNA synthesis (27, 30). Consistently, knockdown of eRNAs leads to decreased expression of nearby target genes (31, 32).

## The Role of eRNAs in Enhancer Function

**Figure 2:**
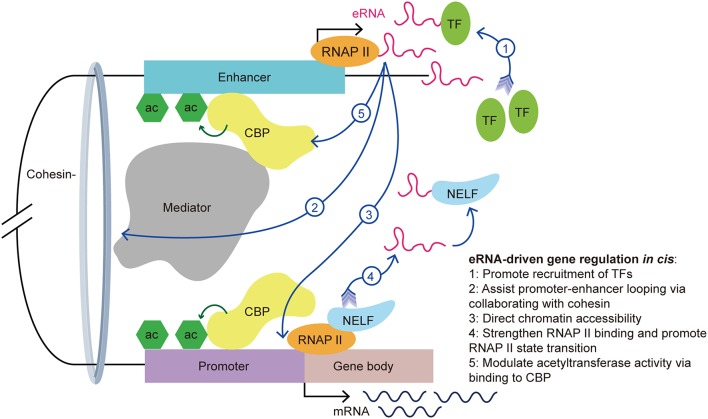
eRNA-driven gene regulation *in cis*. *Cis*-regulatory elements, distal enhancers, and proximal promoters interact with transcription-associated proteins by forming an enhancer-promoter loop. The looping conformation brings the eRNA near to the promoter of the target gene, allowing it exert its function *in cis*. eRNAs exert their gene regulation function via interaction with a variety of transcription-associated proteins, including transcription factors, cohesin, mediators, RNAP II, and CBP.

The actions of eRNAs have been widely studied in recent decades and several functional mechanisms have been proposed. The biological functions of eRNA are associated with TF recruitment, enhancer-promoter looping, chromatin conformation, and histone acetylation (Figure 2) (7–12, 33, 34). In addition to their functional contributions, eRNAs are also markers of enhancer activity. As an independent indicator of enhancer activity, the presence of eRNAs can distinguish whether the enhancer is active or silent. In macrophages, nearly all SEs express seRNAs (93.3%) within intergenic regions, which indicated that the presence of seRNAs could be used to mark SEs (35).

First, recent studies showed that eRNAs can promote the recruitment of TFs. YY1 is a TF that not only binds to active enhancers and promoters, but also binds to eRNA and RNA transcribed from promoters in murine embryonic stem cells. Further investigations revealed that YY1 binding to DNA is stabilized by an eRNA that is tethered by RNAP II (23). It is possible that the eRNA captures free YY1, which allows this TF to bind to a nearby DNA locus. This creates a positive feedback loop in the stimulation of local transcription and allows more TFs to bind the genomic locus. Recently, Weintraub et al. found that YY1 could form dimers and bind to enhancers and promoters to facilitate enhancer-promoter looping (36), which suggested an indirect facilitation effect of the eRNA on enhancer-promoter looping. Similarly, Charles et al. found a novel group of interferon gamma (*IFNG*) eRNAs that bind nuclear factor kappa B (NF-κB) to enhance its function. Treatment of chromatin with ribonuclease led to decreased NF-κB binding to the *IFNG* genomic sites. Using cell transfection techniques, the authors illustrated that knockout of *IFNG-R-49*, an *IFNG* eRNA, resulted in the reduction of NF-κB binding to the *IFNG-D-49* genomic site, which demonstrated that *IFNG* eRNAs contribute to maintaining the binding between NF-κB and the *IFNG* locus (7).

Second, Amartya et al. identified a significant correlation between promoter-enhancer looping, the presence of eRNAs, and gene expression, which suggested that eRNAs are involved in the interaction between enhancers and promoters (37). As part of the gene regulatory mechanism, enhancer-promoter looping is necessary for gene activation (38). A previous study showed that enhancer-promoter looping was modulated in part by the mediator complex and cohesin (21). Following the binding of the mediator complex and cohesin to the enhancer and promoter, looping of the enhancer and promoter brings the eRNA close to the target gene promoter to allow coordination and activation. Knockdown of specific eRNAs reduced enhancer-promoter looping and limited the interplay between transcription effectors that are located within the loop, such as mediator 1, P300 and early growth response 1 (8, 31, 39, 40). Knockdown of the growth regulating estrogen receptor binding (GREB) eRNA led to suppression of enhancer-promoter looping and inhibition of *GREB1* gene induction. Further investigations showed a reduction in cohesin recruitment after eRNA knockdown. This finding suggested that eRNAs promote enhancer-promoter looping via collaborating with cohesin (33).

Third, eRNAs also contribute to directing chromatin accessibility and thus promote specific gene expression. Mousavi et al. identified two seRNAs transcribed from CE and DRR enhancers in MYOD1, a recently labeled SE, in skeletal muscle satellite cells. Depletion of these eRNAs caused reduced chromatin accessibility and RNAP II occupancy at the *MYOD1* and *MYOG* (Myogenin) loci, respectively. Normally, the *MYOG* locus remains inaccessible to nucleases, and chromatin remodeling is needed for the transcription activation of this locus. Using deoxyribonulcease I (DNase I) accessibility as an indicator for remodeling, the authors detected a reduction of DNase I accessibility at specific loci in eRNA-depleted cells. Additionally, they hypothesized that eRNAs are involved in regulating the assembly of transcriptional systems by observing that eRNAs affects RNAP II residency at target genes (9).

Fourth, studies have suggested that eRNAs exert various roles in the interaction with RNAP II. For example, eRNAs strengthen the binding of RNAP II to enhancer regions and promoters (9). Maruyama et al. disclosed the attenuation of diethyl maleate-induced RNAP II binding to promoters in eRNA knockdown cells (41). Moreover, eRNA promotes the paused RNAP II transition into the gene body by acting as a decoy. *Arc* eRNA depletion resulted in a decrease in the elongating form of RNAP II, which indicated that eRNAs promote the state transition of RNAP II. This hypothesis was further supported by the finding that knockdown of *Arc* eRNA led to maintenance of the negative elongation factor complex (NELF) on the promoter. NELF induces RNAP II pausing by binding to RNAP II, the promoter, and the newly generated RNA. Katie et al. suggested that eRNAs bind to NELF via competing with the nascent RNA, leading to the detachment of NELF from RNAP II, thereby enabling RNAP II elongation and mRNA synthesis (11). The state transition of RNAP II from paused to productive elongation is extremely important for target transcript production.

Last but not the least, eRNAs can bind to CBP and modulate the acetyltransferase activity at the enhancer, thus increasing the transcription of target genes. CBP binding to P300 and the resulting high levels of H3K27ac, are hallmarks of enhancers. Having noticed that there was more active transcription from loci with CBP bound to eRNAs than from the no-RNA binding control CBP binding sites, Daniel et al. found that this effect is stimulated by eRNAs binding to the histone acetyltransferase (HAT) domain of CBP. This domain determines the HAT enzymatic activity and this process promotes CBP acetyltransferase activity. The authors demonstrated that eRNA binding exposes the activation loop in CBP/P300. Thus, there is an increase of H3K27ac and H3K18ac at the enhancer and promoter, which increases transcription of the target genes (12). Taken together, eRNAs stimulate target gene transcription in part by stimulating histone acetylation.

## How Super-Enhancer RNAs Regulate Gene Expression: *Cis* Regulation and *trans* Regulation

The functions of seRNAs can be classified as *cis*-regulation and *trans*-regulation. The *cis* regulation by eRNAs has been widely accepted, in which the enhancer-promoter looping structure brings the eRNA close to the target gene. Using chromosome conformation capture (3C) technology, this looping model has been supported by a wide range of studies. As previously discussed, the *cis*-acting function of eRNAs is accomplished via their dynamic interactions with TFs, modifiers, and cohesin subunits within or near the enhancer-promoter loop. Depletion of seRNAs from distal super enhancers at the *NANOG* locus led to significantly decreased expression of *DPPA3* (encoding developmental pluripotency associated 3). *DPPA3* is the nearest gene to the *NANOG* super enhancer apart from *NANOG* itself. Using 3C, the authors demonstrated that the looping of the distal super-enhancer at the *DPPA3* promoter decreased by ~50%, suggesting that the distal seRNA stabilizes the looping and chromatin interactions *in cis*, thereby regulating the expression of *DPPA3* (31). Similarly, the transcription of another seRNA, named *CARMEN* (Cardiac mesoderm enhancer-associated non-coding RNA), was found to cause activation the expression of direct downstream genes (42). Taken together, these results demonstrated that seRNA functions as a scaffold that guides TFs and looping-associated protein complexes *in cis*.

**Figure 3:**
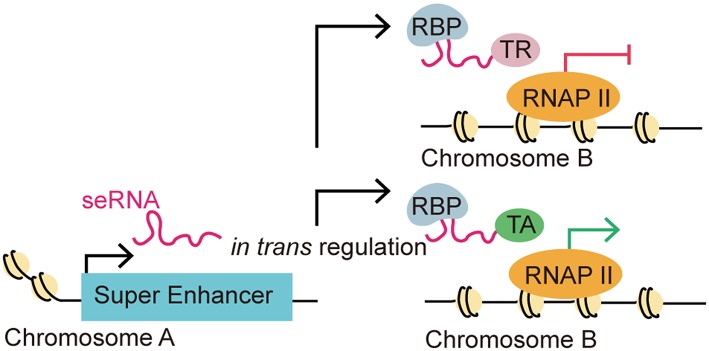
eRNA-driven gene regulation *in trans*. Recent studies identified that eRNAs could regulate gene transcription *in trans*, which means that an eRNA could affect gene regulation in a distal target gene in a different chromosome. This distal regulation function is accomplished via interactions with RNA-binding proteins (RBPs) and other transcription-associated proteins. The *trans* regulation by eRNAs can be classified as repressive or activating depending on their interaction with transcription repressors (TRs) or transcription activators (TAs).

Intriguingly, some recent studies suggested seRNAs might also function interchromosomally (*trans* activity) to direct target gene expression (Figure 3) (8, 25, 32, 43). For instance, the MYOD Upstream Non-coding RNA (*MUNC*), an eRNA originating from the distal regulatory region enhancer of *MYOD*, was observed to induce the transcription of specific myogenic genes [e.g., *MYOD, MYOG*, and *MYH3* (myosin heavy chain 3)] *in trans*. Overexpression of *MUNC* in *MYOD*^−/−^ cells caused *MYOG* and *MYH3* transcription and expression. Notably, these two genes are located on different chromosomes, validating MUNC's *trans* activity (32). Alcarez-Dominguez et al. reported that a polyadenylated-eRNA-producing Band 3 SE transcribes an seRNA called *Bloodlinc* that can facilitate gene expression and stimulate red cell production *in trans* (25). Strikingly, they found that *Bloodlinc* diffused beyond its domain of transcription and 81 direct gene targets located across multiple chromosomes were identified as regulated reciprocally upon *Bloodlinc* depletion or overexpression. This is quite different from typical eRNAs, which usually remain in proximity to their parent enhancers. Many of the regulated genes were located outside the super-enhancer domain. Further investigations showed that *Bloodlinc* binds to *trans*-chromosomal loci that encode key erythroid modulators and TFs. Using mass spectrometry [comprehensive identification of RNA-binding proteins by mass spectrometry (ChiIRP-MS)] techniques, the authors found that *Bloodlinc* interacted with multiple protein complexes that function as RNA helicases (e.g., DExD-box helicase 21), RNA transporters (e.g., heterogeneous nuclear ribonucleoprotein A1), RNA splicers (e.g., KH-type splicing regulatory protein), chromatin organization regulators (e.g., marker of proliferation Ki-67 (MKI67), and Lamin A/C), and transcription coactivators or co-repressors [e.g., MYB binding protein 1a (MYBBP1A) and heat shock protein family A member 8 (HSPA8)]. Moreover, immunoprecipitates of endogenous heterogeneous nuclear ribonucleoprotein U (HNRNPU), a nuclear matrix protein that stabilizes RNA-chromatin associations, were specifically enriched for *Bloodlinc*, which confirmed the interaction between *Bloodlinc* and HNRNPU (25). Thus, these findings suggested a model for how *Bloodlinc* acts *in trans*. Specifically, *Bloodlinc* accesses its *trans* target genes via chromatin interactions stabilized by HNRNPU. *Bloodlinc* stimulates or represses target genes expression via interacting with transcription coactivators (e.g., MYBBP1A) or transcriptional repressors (e.g., HSPA8). This process is stabilized by chromatin organization regulators that also interact with *Bloodlinc*.

## Cancer Associated Super Enhancer RNA

To determine whether enhancers or eRNAs correlate with disease-associated DNA sequence variations, Hnisz et al. investigated the distribution of SNPs within enhancers and super-enhancers. They found that SNPs were enriched in enhancers and SEs, with trait-associated SNPs occurring in SEs at a strikingly higher rate than in enhancers. Analysis in a colorectal cancer cell line demonstrated that more than one-third of SE genes have functions that are closely related with cancer hallmarks, such as evading growth suppressors (e.g., Cyclin D1, Epiregulin), avoiding immune destruction (e.g., F2R like trypsin receptor 1) and sustaining proliferative signaling (e.g., insulin receptor substrate 1, KIT ligand) (2, 16). Consistently, other studies found that SEs are associated with critical tumor oncogenes in various types of cancers. Lovén et al. found that in multiple myeloma, disruption of *BRD4* (bromodomain-containing Protein-4) and mediator occupancy in an SE led to inhibition of tumor oncogenes, including MYC (44). Recently, eRNAs were found to participate in regulating gene transcription and cell-cycle progression with *TP53* (p53 tumor suppressor). Melo et al. found some of the *TP53* binding regions encompass enhancer activity and produce eRNAs in a p53-dependent manner. Knockdown of these eRNAs significantly inhibited downstream target gene transcription upon *TP53* activation, suggesting the eRNAs produced from *TP53* bound enhancer regions that are required for efficient *TP53* transcription enhancement and p53-dependent cell-cycle arrest (45). Moreover, Jiao et al. found a heparanase eRNA that is elevated in cancer cell lines, and enhances tumorgenesis and aggressiveness of cancer cells by facilitating chromatin looping between a super enhancer and the *HPSE* (heparanase) promoter (39). The results from these studies indicated that seRNAs might have significant roles in tumorgenesis and could serve as potential targets for cancer clinical therapy.

## Immune Checkpoint

Immune checkpoints, including stimulatory and inhibitory pathways, are regulatory signals that play vital roles in the maintenance of the delicate balance between activation of adaptive immunity and retaining self-tolerance from autoimmunopathy. Stimulatory checkpoint molecules encompass CD28 and the inducible T cell costimulator (both from the B7-CD28 superfamily) as well as CD27, CD40, OX40 (TNF receptor superfamily member 4), and CD137 (TNF receptor superfamily member 9), which are all from the tumor necrosis factor (TNF) receptor superfamily. Inhibitory checkpoint molecules include members of the B7-family [such as CTLA-4 (CD 152), PD-1 (CD 279)], Lymphocyte Activation gene-3, and nicotinamide adenine dinucleotide phosphate. Through unique and non-redundant pathways, these molecules work as secondary signals to determine the activation or inhibition of immune cells upon antigen recognition, which modulates the duration and amplitude of physiological immune responses.

Cancer cells are capable of evading immune recognition and immune-mediated destruction by downregulating the expression of tumor antigens, seizing inhibitory immune checkpoints, and inducing immune exhaustion, which leads to the increased expression of inhibitory receptors on T cells, such as CTLA-4 and PD-1 (16). Exhausted T cells often feature CTLA-4 expression. CTLA-4, as a B7/CD28 family member, is involved in tumor immune evasion via down-regulation of CD4^+^ effector cells (T_eff_) and promotion of T_reg_ cell activity (13). PD-1, another marker of T cell exhaustion, is expressed at a characteristically high level in tumor infiltrating T cells, which is in consistent with a reduction in interleukin (IL)-2 and IFNγ production and cell cycle arrest in T cells (46). In addition, tumor cells and tumor associated antigen presenting cells (APCs) often express higher levels of co-inhibitory molecules than co-stimulatory molecules, which enhances the activation threshold of T cell and leads to T cell anergy (47).

Rheumatic diseases are characterized by abnormal activation of the immune system, which leads to chronic inflammation and tissue damage. Immune checkpoints are actively involved in the manifestation of rheumatic disease. Genetic polymorphisms in the PD-1 gene (*PDCD1*) in humans correlate with a variety of autoimmune diseases, including type 1 diabetes (T1D), rheumatoid arthritis (RA), multiple sclerosis, and systemic lupus erythematosus (SLE) (48). Mice deficient for a single inhibitory receptor (such as CTLA-4 or PD1) often display enhanced susceptibility to experimentally-induced autoimmune diseases or may spontaneously develop a lupus-like disease (49). CTLA-4 has a fundamental role in establishing immune tolerance. *Ctla4* knockout mice showed premature death caused by the development of lymphoproliferative disease with multiple organ involvement (50), while human patients with mutations that caused loss of function of CTLA-4 also manifested widespread immune dysregulation (51). Jury et al. identified CTLA-4 dysfunction as a possible cause of abnormal T-cell activation in patients with SLE (52). In addition, autoimmunity activation and inflammatory toxicities, such as colitis, hepatitis, pneumonitis, dermatitis, and myasthenia gravis, are major adverse events caused by the use of immune-checkpoint blockers in tumor immunotherapy (53–56). The use of Ipilimumab, a CTLA-4-blocking antibody, was reported to cause inflammatory exacerbation in 25% of patients who had preexisting autoimmune diseases (57). This led to the hypothesis that enhancing inhibitory pathways would be beneficial to treat autoimmune disease. Abatacept, a CTLA4–Fc fusion protein, is the first checkpoint-targeting drug to be approved to treat rheumatic diseases. CTLA-4 Fc prevents costimulatory signaling, thus reducing T cell activation in RA, SLE, and psoriatic arthritis (58–60).

PD-1 and CTLA-4 are the most widely studied inhibitory checkpoint molecules because of their superior performance in the treatment of tumors (Table 1). PD-1 is expressed on T cells, B cells, dendritic cells (DCs), monocytes, natural killer (NK) T cells, exhausted cells, and T_reg_ cells. When engaged to its receptor PD-L1, which is widely expressed on antigen-presenting cells, CD4^+^ T cells, and non-lymphoid tissues, PD-1 delivery an inhibitory signal via direct and indirect pathways. In the direct pathway, PD-L1-engaged PD1 potently counteracts CD28-co-stimulation and T cell receptor (TCR) signal transduction via terminating zeta chain of T cell receptor associated protein kinase 70 and phosphoinositide-3-kinase (PI3K) phosphorylation, leading to recruitment of protein tyrosine phosphatase non-receptor type 11 (PTPN11), which in turn inhibits IL2 production and reduces the transcription of pro-survival factor BCL-2-like protein 1 (73). In the indirect inhibitory mechanism, engaged PD1 decreases casein kinase 2 alpha 1 expression and activity, which results in the maintenance of phosphatase and tensin homolog activity, shutting off both the protein kinase B (AKT) pathway and subsequent T cell growth (73). In addition, the inhibitory function of PD-1 is exemplified by the promotion of TCR endocytosis and shifting the metabolic status of T cells toward fatty acid beta-oxidation, which leads to metabolic restriction (73). While CTLA-4 plays a pivotal role in attenuating the activation of naïve and memory T cells via competing with CD28-mediated signaling (74). Downstream of both CTLA-4 and PD-1 abrogates AKT activity, which is related to limiting cellular metabolism (70).

**Table 1:** Overview of PD-1/PD-L1 and CTLA-4 blockage.

**Molecule**	**Ligand (s)**	**Expressing cells**	**Blockage approved for**	**Blockage effects**	**References**
PD-1	PD-L1, PD-L2	T cells, NK cells, B cells, macrophages, DC subsets, mast cells	Metastatic melanoma, non-small-cell lung cancer, head and neck squamous cell cancer, Hodgkin's lymphoma, renal cell carcinoma	Restore TCR signaling. Enhance IFN-γ and associated chemokines. Promote CD8^+^ T cell influx in tumor microenvironment. T cell metabolic reprogramming	(61–66)
PD-L1	PD-1, B7-1 (CD80)	Tumor cells, tumor-associated APCs (e.g., DC, monocytes, macrophages, mast cells, T cells, B cells, NK cells)	Non-small-cell lung cancer, bladder cancer, urothelial carcinoma, Merkel cell carcinoma	Target:Cancer cell: Block PD-1/PD-L1 signaling pathway. Block interaction with CD80. Inhibit immune-independent cancer cell intrinsic growth. Macrophage: •Suppress T cell extrusion from tumor microenvironment	(66–69)
CTLA-4	CD80, CD86	T cells (resting and activating)	Metastatic melanoma, renal cell carcinoma	Block competitive inhibition of CD28 co-stimulation. T cell metabolic reprogramming Broaden the peripheral TCR repertoire	(70–72)

## Clinical Implications of Immune Checkpoints

Immunotherapy, especially PD-1/PD-L1 blockage and CTLA-4 blockage, has revolutionized the landscape of cancer treatment in recent years. The FDA has approved immune checkpoint inhibitors for the treatment of a range of tumor types, such as melanoma, non-small cell lung cancer, renal cell carcinoma, Hodgkin lymphoma, and head and neck squamous cell carcinoma.

### PD-1/PD-L1 Blockage: Mechanism and Effect

By counteracting the pathological function of PD-1, antibodies that block PD-1 (e.g., Pembrolizumab and Nivolumab) and its ligand PD-L1 (e.g., Atezolizumab, Avelumab, and Duralumab) inhibit adaptive immune resistance and reinvigorate the immune response against cancer cells. PD-1 mediates immune suppression via a variety of mechanisms in cancer. In T cells, PD-L1-bound PD-1 inhibits TCR signaling by recruiting PTPN11 to the immunoreceptor tyrosine-based switch motif domain, which results in dephosphorylation of downstream signaling molecules, decreased IL-2 production, reduction in cell cycle progression, and reduced expression of TFs involved in effector function (T-bet and eomesodermin) (74). An elevated level of circulating IFNγ and its associated chemokines [C-X-C motif chemokine ligand (CXCL)-9 and CXCL-10] and T cell activation markers (MKI67 and major histocompatibility complex, class II, DR) were detected in the serum of patients undergoing anti-PD-1 and anti-PD-L1 treatment (61). PD-1 blockage restores T cell activation and an influx of CD8^+^ T cells was detected in the tumor microenvironment (62). In addition, PD-1 signaling interferes with CD28-mediated activation of PI3K and AKT, which in turn limits glucose metabolism (70). The resulting bioenergetic insufficiencies inhibit mammalian target of rapamycin (mTOR) activity and IFNγ production, impair EZH2 (enhancer of zeste 2 polycomb repressive complex 2 subunit) expression in T cells, and reduce the level of phosphoenolpyruvate, which is linked with a lack of activation of CD4^+^ tumor-infiltrating lymphocytes (61). The process of metabolic restriction is a driver of T cell exhaustion. Antagonists of PD-1 cause T cell metabolic reprogramming and restore their glycolytic capacity, as well as the subsequent effector function (63). PD-1 and PD-L1 blockage also decrease E3 ubiquitin ligase CBL-B expression thus inhibiting the downregulation of TCR (64).

PD-L1 expression is especially high in tumor cells and tumor-associated APCs (e.g., tumor environment DCs, macrophages, and fibroblasts) (75). As a result of adaptive immune resistance, PD-L1 overexpression on tumor cells is induced by IFNγ that is produced by activated T cells. High levels of PD-L1 expression have been associated with poor prognosis in many types of cancer. PD-L1 antibodies exert their antitumor effect partly by blocking the PD-1–PD-L1 interaction. Manish et al. found that PD-L1 also interacts with B7-1 (CD80) to inhibit T cell activation and proliferation (67). Therefore, PD-L1 blockage also may restore T cell activation by inhibiting the CD80–PD-L1 interaction. A recent study showed that tumor-expressed PD-L1 has tumor-intrinsic effects in addition to delivering an inhibitory signal to PD-1 on T cells. PD-L1 promotes cell-intrinsic growth in an immune-independent manner in both melanoma and ovarian cancer. PD-L1 represses tumor autophagy and enhances the mTOR pathway in both ovarian cancer and melanoma (68). Thus, PD-L1 blockage may exert its effect by mediating PD-L1-related intrinsic tumor signaling. PD-L1 expression on macrophages may result in active extrusion of T cells from the tumor microenvironment, indicating another possible pathway for PD-L1 blockage (69).

## Crosstalk Between seRNAs and Immune Checkpoints

Super-enhancers play a critical role in the regulation of genes that define cell identity, and increasing evidence suggests that SEs and eRNAs have functions in tumorgenesis. However, the exact function and mechanism of seRNAs in the regulation of tumorgenesis and tumor immunotolerance is not fully understood. Lovén et al. found that cancer cells acquire SEs at oncogenes and at genes that are important for tumor pathogenesis. They found that SEs assist the high level transcription of genes [e.g., *MYC, IRF4* (interferon regulatory factor 4), *XBP1* (X-box binding protein 1), *CCND2* (Cyclin D2)] that are deregulated in multiple myeloma cells (44). Ding et al. revealed the oncogenic role of eGREB1, an eRNA of an estrogen-responsive gene enhancer, growth regulating estrogen receptor binding 1 (GREB1), in bladder cancer. Upregulated eGREB1 is associated with higher level TNM stages of bladder cancer. Consistently, proliferation, migration, and invasion were inhibited upon eGREB1 knockdown, while apoptosis was promoted (76). In addition, for a variety of cancer cells (e.g., pancreatic cancer and T cell leukemia), SEs were identified around the *MYC* gene in cancer cells. However, these SEs were not identified in their healthy counterparts (2). Besides, Hnisz et al. provided a list of tumor-specific SEs that fall into different categories of hallmark cancer genes in colorectal cancer. For instance, the identified SEs of *PCDH7* (protocadherin 7), *CCND1* (Cyclin D1), and *F2RL1* are associated with activating invasion and metastasis, evading growth suppressors, and avoiding immune destruction, respectively (2). Taken together, these findings indicated that SEs might act as keys for amplified oncogene expression. This hypothesis was supported by the results of Wong et al., who found that multiple oncogenic TFs are regulated by SEs in acute T cell lymphoblastic leukemia. Disruption of SE-related gene expression and cancer cell death were identified after treating cancer cells with RNAP II activation blocker (77). SeRNAs play an active role in promoting SE function; therefore, we suspected seRNAs could also function in this inhibitory process.

### Super-Enhancer RNA Induces PD-L1 Expression via Enhancing MYC Expression

*MYC* is an oncogene that has been studied in depth. The activation and overexpression of *MYC* is a characteristic feature of tumorgenesis and cancer maintenance. As a TF, MYC activates the expression of many pro-proliferative genes by binding at enhancers and recruiting HATs (78). One of the mechanisms by which the *MYC* gene is believed to maintain cancer cell survival is to exempt itself from immune surveillance and the anti-tumor immune response (79). This hypothesis was supported by the finding that *MYC* expression correlated highly with *PDCD1L1* (PD-L1) gene expression in non-small cell lung cancer cell (79). Kim et al. also identified poorer clinical outcome for patients with both MYC and PD-L1 dysregulation and overexpression (79). Consistently, Casey et al. demonstrated that MYC upregulates the expression of immune checkpoints, *CD47* and *PDCD1L1*, on cancer cells by direct interaction with the promoters of these two genes. In multiple types of cancer, silencing of *MYC* leads to a significant reduction in the transcription and expression of *CD47* and *PDCD1L1*, both *in vitro* and *in vivo* (80). Therefore, MYC may be a key regulator for immune checkpoint expression in cancer. For a variety of cancer cells, SEs are found specifically clustered at genes surrounding the *MYC* gene (2). SEs tend to express seRNAs at higher levels than typical enhancers; therefore, we wondered whether seRNAs participate in the regulation of immune checkpoint expression. Human colorectal cancer-specific nucleus retained Colorectal Cancer Associated Transcript 1-long isoform (CCAT1-L) is a 2,600 nucleotide long lncRNA that is transcribed from an SE and therefore is considered as an seRNA. A recent study suggested that the seRNA CCAT1-L contributes to the regulation of *MYC* expression *in cis* in colorectal cancer. CCAT1-L is transcribed from a locus 515 kb upstream of the *MYC* gene (MYC-515) in the human 8q24 region. This SE forms an enhancer-promoter loop with the *MYC* promoter, thus bringing CCAT1-L in to close proximity with the promoter. Such chromatin interactions are present specifically in colorectal, breast, and prostate cancer (81). Xiang et al. reported that CCAT1-L assists in the maintenance of chromatin looping between the SE and the *MYC* promoter (82). Previous studies revealed that eRNAs participate in gene regulation by stabilizing enhancer-promoter looping and by dynamic interactions with TFs and mediators in the surrounding area. The accumulation of CCAT1-L surrounding its SE indicates its possible function in the regulation of its target gene. By examining the expression of mRNA after CCAT1-L knockdown, reduced *MYC* transcription and expression were detected. However, overexpression of CCAT1-L from a plasmid showed no apparent activation of *MYC* expression. This could be explained by the possibility that extrinsic CCAT1-L localized to many nuclear sites but not its *in cis* site. To confirm this hypothesis, Xiang et al. (82) used transcription activator-like engineered nucleases to achieve *in cis* overexpression of CCAT1-L in a low CCAT1-L expression cell line. CCAT1-L overexpression resulted in higher *MYC* expression and faster cell growth than that in the control cancer cell group. Thus, CCAT1-L enhances *MYC* expression *in cis*. The most common form of seRNA function is *in cis* regulation; however, how is CCAT1-L brought close to its target gene from 515 kb away? To answer this question, Xiang et al. applied the 3C technique to investigate the interaction frequencies between possible enhancers and *MYC* promoter segments. Intriguingly, they found an interaction between a locus 335 kb upstream of the *MYC* promoter (MYC-335), and the *MYC* promoter showed the highest interaction frequency with this site, while the interaction between MYC-515 and MYC-355 ranked as second. Earlier Pomerantz et al. found an enhancer located at MYC-355, which forms a loop between the MYC promoters to promote its transcription (83). The result of 3C analysis suggested that CCAT1-L locates to MYC-335, bringing it closer to *MYC*. Knockdown of CCAT1-L resulted in a prominent decrease in the chromatin interactions between MYC-335 and the *MYC* promoter and between MYC-515 and MYC-335. Taken together, the results suggested that a looping conformation is formed between *MYC* and MYC-335 and between MYC-335 and MYC-515. In addition, CCAT1-L is required to maintain the specific loops between the *MYC* enhancers and the *MYC* promoter. Further investigation into the exact mechanism by which CCAT1-L functions to promote enhancer-promoter looping revealed that TFs transcription factor 4 and CCCTC-binding factor (CTCF) are enriched at the loops of the *MYC* promoter, and the MYC-335 and MYC-515 segments. Moreover, knockdown of *CTCF* is associated with decreased transcription of *MYC* and CCAT1-L, suggesting that the enhancer-promoter looping at *MYC* is CTCF-mediated. In addition, RNA immunoprecipitation showed a specific interaction between CTCF and CCAT1-L. Reduced CTCF occupation of the loop region at *MYC* was detected after depletion of CCAT1-L, indicating that CCAT1-L assists the binding of CTCF to chromatin and contributes to the looping formation at the *MYC* locus (82). In summary, Xiang et al. demonstrated the involvement of seRNA CCAT1-L in the regulation of key oncogene *MYC* in colon rectal cancer. CCAT1-L regulates the expression of *MYC* by interacting with CTCF and assisting its binding with chromatin to sustain the enhancer-promoter looping conformation between the *MYC* promoter and MYC-335 and between MYC-335 and MYC-515. *MYC* expression has been linked to the regulation of a variety of cancer hallmark-related genes in tumors. As mentioned above, MYC upregulates the expression of immune checkpoints *CD47* and *PDCD1L1* on cancer cells by direct interaction with promoters of these two genes. Therefore, it is possible that seRNA CCAT1-L participates in the regulation of *CD47* and *PDCD1L1* expression indirectly by promoting *MYC* gene expression *in cis*.

**Figure 4:**
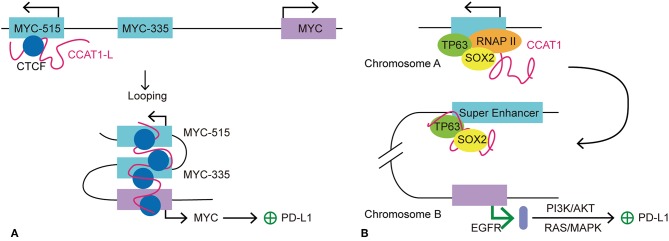
Models of seRNA CCAT1 in the regulation of PD-L1 expression. CCAT1 is an oncogenic seRNA that can regulate the expression of PD-L1 via *cis* and *trans* actions. **(A)** SeRNA CCAT1-L induces the expression of PD-L1 via enhancing the expression of *MYC in cis*. With the help of CTCF, CCAT1-L is brought close to the *MYC* promoter via MYC-515-MYC-335-*MYC* looping. This looping conformation promotes *MYC* transcription, which allows its further promotion of PD-L1 expression. **(B)** TP63 and SOX2 are two transcription factors that promote seRNA CCAT1 transcription. CCAT1 interacts with TP63 and SOX2 to further act *in trans* on another chromosome to promote the transcription of *EGFR*. EGFR promotes PD-L1 expression via its downstream pathways, PI3K/AKT and RAS/MAPK.

Additionally, Jiang et al. recently demonstrated that CCAT1 interacts with the TFs tumor protein p63 (TP63) and SRY-box 2 (SOX2) to regulate the expression of the epidermal growth factor receptor (EGFR) in esophageal squamous cell carcinoma (ESCC) (84). With identification of TP63 and SOX2 co-occupied at an SE, the authors wondered whether there is interplay between the seRNA and TP63 and SOX2. Further investigation showed that transcription of seRNA CCAT1 is activated or inhibited by TP63 and SOX2 co-binding or depletion at the promoter and SE of CCAT1. Thus, seRNA CCAT1 was validated as the downstream interaction target for TP63 and SOX2. Moreover, seRNA CCAT1 forms a complex with TP63 and SOX2 and then binds to SEs of *EGFR* to enhance its transcription and expression. The transcription of *EGFR* activates the downstream PD-L1 expression related pathways, including RAS/mitogen activated protein kinase (MAPK) and PI3K/AKT signaling pathways (84). Consistently, the expression of PD-L1 is elevated significantly in response to EGFR signaling activation in esophageal squamous cancer cells. By contrast, inhibition of the EGFR pathway led to a sharp decline in PD-L1 expression (85, 86). Further investigation showed that EGFR-dependent expression of PD-L1 in ESCC is affected by EGFR/PI3K/AKT, EGFR/RAS/MAPK and EGR-phospholipase C gamma 1 (PLC-γ) signaling pathways (84). Many studies have revealed the close connection between PD-L1 expression and *EGFR* mutation (87–89). Higher PD-L1 expression usually indicates poor prognosis (88, 89). For instance, in non-small-cell lung cancer, mutation in *EGFR* lead to upregulation of PD-L1 by activating PI3K/AKT and RAS/extracellular signal-regulated kinase (ERK) signaling (90). Notably, the *EGFR* gene is located in the 7p11 region in the human genome, indicating that CCAT1 promotes *EGFR* transcription *in trans*. Taken together, we propose that seRNA CCAT1 could be involved in the regulation of *PDCD1L1* transcription *in trans* by forming a seRNA-TF complex to promote *EGFR* expression and activate the downstream signaling pathways (Figure 4).

CDK7 affects transcription initiation and elongation by blocking SE normal function. As for its potential clinical application, Chipumuro et al. found cyclin-dependent kinase 7 (CDK7) inhibitor, THZ1, selectively downregulates SE-regulated *MYCN* overexpression and MYCN-driven transcription amplification in neuroblastoma (91). Similarly, other researchers revealed THZ1 suppressed SE-driven oncogenic transcriptional amplification in other cancers (92, 93).

### Relationship Between BRDs, seRNAs, and PD-L1

**Figure 5:**
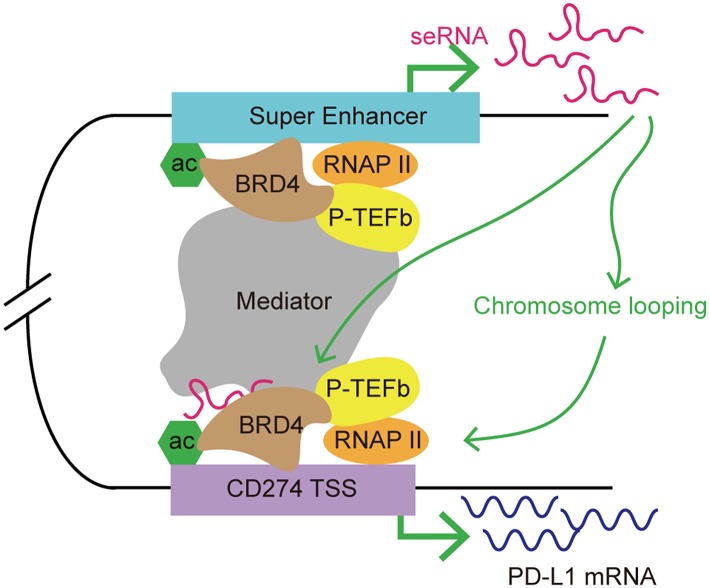
A model of seRNA in the regulation of PD-L1 expression via BRD4. BRD4 is enriched at super enhancers and contributes to seRNA transcription. *PDCD1L1* (also known as *CD274*; encoding PD-L1) is a direct target of BRD4. BRD4 recruits mediator and P-TEFb to acetylated chromatin, leading to the activation of gene expression via phosphorylating RNAP II. An seRNA transcribed from a distal super enhancer proximal to *CD274* is found to loop with the *CD274* transcription start site (TSS). This seRNA enhances the chromosome looping stability and interacts with BRD4 to promote CD274 expression.

BRD4 is a member of BET family, which includes BRD2, BRD3, and bromodomain testis-specific proteins (BRDT). As a transcription co-activator, BRD4 is often required for the expression of oncogenes, including *MYC* (94–96). The functions of BET proteins include initiation and elongation of transcription, and cell cycle control. BRD4 recruits a variety of transcription complexes, including mediator and positive transcriptional elongation factor b (P-TEFb), to acetylated chromatin, leading to the activation of gene expression via phosphorylation of RNAP II (97, 98). Studies showed accumulation of BRD4 at SEs, which facilitated eRNA transcription via interacting with acetylated histones to assist RNAP II progression (99). Recently, BET inhibitors (i.e., JQ1) have been developed for anticancer treatment. JQ1 displaces BRD4 from chromatin, breaks the cell cycle, and induces apoptosis in tumor cells. JQ1 also inhibits BRD4-associated seRNA synthesis by targeting SEs preferentially (44, 99). When treated with JQ1, SEs displayed a higher level of loss of BRD4 accumulation than typical enhancers (44). eRNAs have a role in enhancer function; therefore, it is possible that BRD4 might regulate target gene expression indirectly via its effect on eRNA synthesis. A recent study demonstrated co-occupancy of BRD4 and mediator at *MYC* SEs, and the use of BET inhibitor JQ1 resulted in transcription elongation defects in *MYC* (44). JQ1 displacement of BRD4 from the *MYC* promoter/enhancer also led to suppression of MYC-driven malignancies, such as multiple myeloma and acute myeloid leukemia (95, 96). Zhu et al. found that treatment with a BET inhibitor suppressed PD-L1 expression in ovarian cancer (100). In a mouse model, the authors observed a dose- and time-dependent reduction in the level of *PDCD1L1* transcription and expression during treatment with JQ1 in tumor cells, macrophages and tumor-associated DCs. Knockdown of *BRD4* decreased PD-L1 expression, indicating that PD-L1 expression is at least partly regulated by BRD4. Further investigation demonstrated that the PD-L1 encoding gene, *PDCD1L1* (also known as *CD274*), is a direct target of BRD4. In addition, the application of JQ1 increased CD8^+^ cytotoxic T cell activity; thus promoting anti-tumor immunity, limiting ovarian cancer progression, and improving mouse survival (101). The mechanism by which BRD4 regulates *PDCD1L1* expression in cancer cell has been determined. Hogg et al. demonstrated that the inhibition of *PDCD1L1* transcription by the BET inhibitor is independent of *MYC* expression, which is usually involved in PD-L1 expression regulation and is also as a target for BET inhibitors in hematologic malignancies (102, 103). The authors found that downregulation of *PDCD1L1* under JQ1 treatment was not associated with changes in *MYC* regulation. Ectopic overexpression of *MYC* did not affect JQ1's suppression of PD-L1 expression. In addition, depletion of BRD4 led to *PDCD1L1* transcription inhibition in the absence of putative changes in *MYC* expression. Using chromatin immunoprecipitation sequencing and analysis of RNAP II occupancy, the authors further identified a distal SE proximal to the *PDCD1L1* gene. Moreover, this distal SE bridged to the *PDCD1L1* transcription start site (TSS), forming a chromatin loop. The accumulation of BRD4 at the *PDCD1L1* TSS decreased substantially upon JQ1 treatment, indicating that disruption of the TSS and SE loop might contribute to JQ1-mediated PD-L1 expression inhibition (103). As mentioned previously, BRD4 promotes eRNA transcription by interacting with acetylated histones. A recent study demonstrated that the BRD4–eRNA interaction promotes the binding of BRD4 to acetylated histone. This interaction further potentiates BRD4 recruitment to enhancers and promotes subsequent transcription events (104). Therefore, we hypothesized that seRNAs might be involved in BRD4-mediated PD-L1 regulation by contributing to stability of the chromatin loop formed by the distal SE and the *PDCD1L1* TSS, which promotes RNAP II progression. BRD4 regulates the expression of *PDCD1L1* indirectly by promoting the transcription of an SE, and the resulting seRNA contributes to promoting enhancer activity, thus affecting the transcription of *PDCD1L1* (Figure 5).

### Super-Enhancer RNA and PD-L1 Expression in Autoimmune Disease

The application of immune checkpoints in oncology sometimes triggers auto-inflammatory adverse effects, which has prompted further investigations of the contribution of immune checkpoints to autoimmunity. The expression and functions of inhibitory immune checkpoints are often dysregulated in autoimmune diseases. Promoting the inhibitory function of immune checkpoints should be beneficial to restore the immune balance in rheumatic disease. However, Farh et al. found that distinct enhancers and eRNAs are involved in autoimmune disease (Table 2). Genome-wide association studies revealed that risk loci for autoimmunity are enriched in immune cell-specific SEs and enhancers (111). Peeters et al. revealed a disease-specific super-enhancer signature in CD4^+^ memory/effector cells in the synovial fluid of patients with juvenile idiopathic arthritis (105). These SEs are associated with inflammatory arthritis SNPs, indicating the contribution of SEs and, possibly, seRNAs in the control of disease pathogenesis. Moreover, the application of BET inhibitors suppressed SE-associated gene expression subsequent to a reduction in proinflammatory markers (105). A previous study found that in T cells, one-third of the non-coding RNAs are transcribed from SEs, indicating their potential role in regulating the T cell immune response (112). In T cells, cytokine receptors and cytokines are the predominant type of genes that have an SE architecture (112). ADAM like decysin 1 (ADAMDEC1) is a member of the ADAMs (A Disintegration And Metalloproteinase) protein family. *ADAMDEC1* and *ADAM28*, which is located upstream of *ADAMDEC1*, are overexpressed in SLE and are upregulated in inflammatory states. Further investigation showed that the interaction between eRNAs and P300 is involved in *ADAMDEC1* expression regulation (106). One of the functions of eRNAs is to bind CBP and modulate the acetyltransferase activity at enhancers. NF-κB is recruited to the enhancer upon activation of the inflammatory signal cascade. The accumulation of NF-κB leads to assembly of P300 on the enhancer, which can be activated by eRNAs. Activation of P300 leads to increased histone acetylation and transcription elongation (106). That study revealed the participation of eRNAs in the regulation of autoimmune-associated gene expression. Therefore, it was suggested that there is crosstalk between immune checkpoints and seRNAs in the context of autoimmunity. BTB domain and CNC homolog 2 (BACH2), a TF that functions to suppress effector programs to maintain the T_reg_-mediated immune homeostasis, has the most prominent super-enhancer in its gene locus in T cells (113). BACH2 regulates the expression of a variety of cytokines in T cells. Genetic variation in the *BACH2* locus is associated with autoimmune-related diseases, such as Crohn's disease, RA, and T1D (114–116). Vahedi et al. found that the *BACH2* locus is SE-regulated, with high P300 occupancy. Knockdown of *BACH2* led to a significant increase in the expression of genes with an SE architecture in T cells, including those encoding cytokines and cytokine receptors (112). In addition, seRNA-related transcription is inhibited by BACH2 (112). Therefore, the authors identified a network in which the expression levels of genes and eRNAs are negatively regulated by BACH2, which itself is also SE-regulated (112). Recent research found that BACH2 promotes tumor immunosuppression via IFNγ and T_reg_-mediated intratumoral CD8^+^ T cell inhibition. Elevated levels of IFNγ were observed in tumors of mice with BACH2 deficiency. Further analysis revealed that suppression of IFNγ is caused by BACH2-mediated T_reg_-dependent tumor immunosuppression (117). Thus, the results demonstrated a pathway for BACH2 to regulate the expression of IFNγ immunosuppression. There are two interferon regulatory factor-1 (IRF-1) binding sites on the promoter of *PDCD1L1*. Diaz et al. revealed that IFNγ signaling is the primarily regulating signal for *PDCD1L1* expression in melanoma cells. Diaz et al. identified the IFNγ-Janus kinase (JAK)-signal transducer and activator of transcription (STAT)-IRF1 axis that regulates PD-L1 expression (118). Such findings link BACH2 with PD-L1 expression, indicating its potential influence on immune checkpoint expression. Similarly, in the context of autoimmune disease, Osum et al. demonstrated that IFNγ drives PD-L1 expression on islet beta cells in T1D (119). T cell-directed beta cell destruction is the main cause of T1D. *In vivo* and *in vitro* experiments showed that T cell infiltration-dependent islet beta cell PD-L1 upregulation is mediated by IFNγ (119). In addition, the increased PD-L1 expression correlated with the level of T cell infiltration and insulitis, indicating that elevated PD-L1 expression is a salvage response to islet destruction (119). These findings led us to hypothesize that SE-regulated TF BACH2 might play a role in regulating the expression of PD-L1 indirectly by mediating the expression of IFNγ in autoimmune disease. Moreover, seRNAs might also contribute to this regulation by promoting SE function. However, little research has been performed on the contribution of seRNAs to the regulation of immune checkpoint expression in autoimmune diseases, and the exact contribution of seRNAs to autoimmune diseases remains poorly understood. Further investigation and more direct evidence are required to reveal the details of the crosstalk between seRNAs and immune checkpoints in autoimmune diseases.

**Table 2:** Involvement of SE in autoimmune diseases.

**Disease**	**Cell type**	**Disease associated SE/seRNA**	**SE or eRNA regulated gene(s)**	**Gene function**	**References**
Juvenile idiopathic arthritis	CD4^+^ memory /effector cells	CTLA4 SE CXCR4 SE	*CTLA4 CXCR4*	Preserve self-tolerance Control chemokine binding receptor expression	(105)
SLE	Monocytes Peripheral blood mononuclear cells	Enhancer1, Enhancer2, eRNA157 PDCD1 enhancer	*ADADMEC1 PDCD1*	Escape inhibition by tissue inhibitor of metalloprotease-3 (TIMP-3). Preserve self-tolerance	(106) (107)
Inflammatory bowel disease	CD14+ cells	IFNG-R-49	Not specified	Control of IL22 and IL26 expression levels	(108)
Multiple sclerosis	THP-1 cells	Vitamin D receptor super enhancers (VSE) 1-3	*ZMIZ1 DENND6B USP2 ASAP2 SEMA6B LRG1*	Leukocyte aggregation, actin filament organization, axon guidance, pro-inflammatory cytokine production regulation	(109)
Autoimmune uveitis	Th1	T-bet SE and T-bet seRNA	*IFNγ, TNF, FASL, ILL8RL*, and *CTLA4*	Inflammatory cell infiltration	(110)

## Conclusions

Current knowledge of immune checkpoints and SEs has increased our understanding of immune checkpoint expression in oncology and autoimmunity. Evading immune destruction is a major hallmark of cancer, suggesting that the expression of inhibitory immune checkpoints could be a critical identity character of tumor cells (16). Super enhancers are clusters of enhancers that facilitate gene expression that is important for cell identity. eRNAs contribute to enhancer function via multiple approaches, including enhancing promoter-enhancer looping, facilitating TF assembly, and promoting RNAP II activation. In the present review, we proposed several ways by which seRNAs contribute to immune checkpoint regulation indirectly by mediating the expression of key genes that regulate immune checkpoint expression, and play critical roles in determining cell identity.

**Figure 6:**
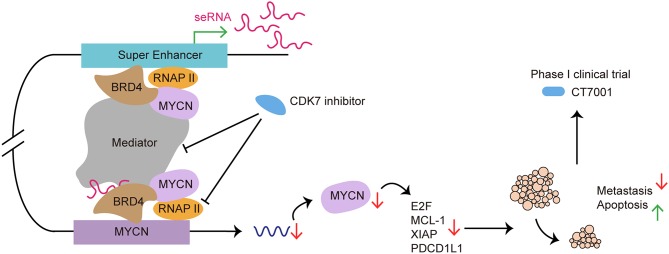
SE as a therapeutic target in cancer treatment: from preclinical to clinical. CDK7 inhibitor targets SE to suppress SE-regulated MYCN driven oncogenic transcription amplification, including *E2F, MCL-1, XIAP* (93). CDK7 inhibitor blocks SE functioning by affecting RNAP II and Mediator complex. Use of CDK7 inhibitor induces tumor regression, cancer cell apoptosis and reduces metastasis both *in vivo* and *in vitro* (120). CT7001, a CDK7 inhibitor, has been approved for phase I clinical trial in patients with advanced malignancies.

As a potential therapeutic target, many researchers had focus on exploring the application of SE blockers in cancers and autoimmune diseases (Figure 6). He et al. recently summarized the role of SE as therapeutic target in cancer treatment. By using BET inhibitor, CDK7 inhibitor, AKT inhibitors, demethylases, and acetyltransferase, researchers target carcinogenic SEs to inhibit cancer growth, invasion, immune escape and progression (121).

Although remarkable process has been made in revealing the regulation of immune checkpoint expression and eRNA function, many questions and challenges remain. For instance, the exact function and mechanism of seRNAs have not been clearly demonstrated. Additional and direct evidence of seRNA function in immune checkpoint expression is required to further support the indirect gene regulation effect by seRNAs. Recent findings also revealed that the exact boundaries between eRNAs and lncRNAs are not absolute, suggesting that some eRNAs might have been mistakenly identified as lncRNAs (28). Despite having a similar frequency of transcription to protein-coding genes, eRNAs have a shorter half-life compared with that of mRNAs and lncRNAs, which represents an obstacle for their thorough study (122). Such characteristics and uncertainty of seRNA make research into the interaction between seRNAs and TFs or chromatin a challenge. Joint efforts should be made by biologists and immunologists to further identify the correlations between SEs, seRNAs, and immune checkpoints. We are only starting to comprehend the full panoply of eRNAs' functions. In the future, a thorough understanding of the mechanism by which seRNAs regulate gene expression and their contribution to disease pathogenesis might help to identify new therapeutic targets and disease biomarkers.

## Author Contributions

All authors listed have made a substantial, direct and intellectual contribution to the work, and approved it for publication.

### Conflict of Interest

The authors declare that the research was conducted in the absence of any commercial or financial relationships that could be construed as a potential conflict of interest.
